# A Delphi consensus: prescribing functional foot orthoses for the symptomatic *pes planus* adult

**DOI:** 10.1186/1757-1146-6-S1-O1

**Published:** 2013-05-31

**Authors:** Helen A Banwell, Karl Landorf, Shylie Mackintosh, Dominic Thewlis

**Affiliations:** 1 International Centre for Allied Health Evidence (iCAHE), School of Health Sciences, University of South Australia, Adelaide, South Australia, 5001, Australia; 2 Department of Podiatry, La Trobe University, Melbourne, Victoria, 3083, Australia; 3 Biomechanics and Neuromotor Lab, Samson Institute for Health Research, University of South Australia, Adelaide, South Australia, 5001, Australia

## Background

Symptomatic (flexible) *pes planus* is a difficult entity to classify with no universally accepted aetiology. Foot orthoses are a commonly used intervention for *pes planus*, however, evidence to support their use is limited. Currently there are no clinical guidelines for the prescription of foot orthoses for *pes planus.* The aim of this study was to seek expert consensus and agreement on prescription paradigms for foot orthoses in the adult symptomatic *pes planus* population.

## Methods

A four round Delphi consensus survey was performed involving 24 podiatric experts from clinical, academic and research backgrounds to establish prescription preferences for the rearfoot, midfoot, forefoot and accommodation variables specific to adult *pes planus*. Round 1 sought individual input with open ended questions (consensus). Rounds 2, 3 and 4 measured individual levels of agreement to statements generated from Round 1 (agreement). Consensus and agreement were pre-determined at 70%.

## Results

Consensus was reached for a single variable (forefoot balance). Agreement was reached for 58 statements involving 25 variables including agreement on when to prescribe: inverted/neutral pour, inverted rearfoot posts, medial heel skives, minimal/standard/maximum arch fill, medial flanges, forefoot posts and other common orthotic accommodations.

## Conclusion

The 26 agreed prescription variables provide a systematically developed expert opinion to base individual prescription choice on in future research involving foot orthoses for people with symptomatic *pes planus* and will aid in the development of prescription guidelines specific to this population.

